# New DNA Methylation Signals for Malignant Pleural Mesothelioma Risk Assessment

**DOI:** 10.3390/cancers13112636

**Published:** 2021-05-27

**Authors:** Giovanni Cugliari, Alessandra Allione, Alessia Russo, Chiara Catalano, Elisabetta Casalone, Simonetta Guarrera, Federica Grosso, Daniela Ferrante, Marika Sculco, Marta La Vecchia, Chiara Pirazzini, Roberta Libener, Dario Mirabelli, Corrado Magnani, Irma Dianzani, Giuseppe Matullo

**Affiliations:** 1 Department of Medical Sciences, University of Turin, 10126 Turin, Italy; alessandra.allione@unito.it (A.A.); alessia.russo@unito.it (A.R.); chiara.catalano@unito.it (C.C.); elisabetta.casalone@unito.it (E.C.); 2 Italian Institute for Genomic Medicine, IIGM, 10060 Candiolo, Italy; simonetta.guarrera@iigm.it; 3 Candiolo Cancer Institute, FPO-IRCCS, 10060 Candiolo, Italy; 4 Mesothelioma Unit, Azienda Ospedaliera SS. Antonio e Biagio e Cesare Arrigo, 15121 Alessandria, Italy; federica.grosso@uniupo.it; 5 Medical Statistics, Department of Translational Medicine, University of Eastern Piedmont, 28100 Novara, Italy; daniela.ferrante@med.uniupo.it (D.F.); corrado.magnani@med.uniupo.it (C.M.); 6 Cancer Epidemiology Unit, CPO-Piemonte, 28100 Novara, Italy; 7 Department of Health Sciences, University of Eastern Piedmont, 28100 Novara, Italy; marika.sculco@uniupo.it (M.S.); marta.lavecchia@uniupo.it (M.L.V.); irma.dianzani@med.uniupo.it (I.D.); 8 IRCCS Istituto delle Scienze Neurologiche di Bologna, 40126 Bologna, Italy; chiara.pirazzini5@unibo.it; 9 Department of Integrated Activities Research and Innovation–Azienda Ospedaliera SS. Antonio e Biagio e Cesare Arrigo, 15122 Alessandria, Italy; rlibener@ospedale.al.it; 10 Cancer Epidemiology Unit, Department of Medical Sciences, University of Turin, 10126 Turin, Italy; dario.mirabelli@cpo.it; 11 Interdepartmental Center for Studies on Asbestos and Other Toxic Particulates “G. Scansetti”, University of Turin, 10126 Turin, Italy; 12 Medical Genetics Unit, AOU Città della Salute e della Scienza, 10126 Turin, Italy

**Keywords:** malignant pleural mesothelioma, asbestos exposure, DNA methylation, epigenome-wide analysis, interaction analysis

## Abstract

**Simple Summary:**

Our study investigated DNA methylation differences in easily accessible white blood cells (WBCs) between malignant pleural mesothelioma (MPM) cases and asbestos-exposed cancer-free controls. A multiple regression model highlighted that the methylation level of two single CpGs (cg03546163 in *FKBP5* and cg06633438 in *MLLT1*) are independent MPM markers. The epigenetic changes at the *FKBP5* and *MLLT1* genes were robustly associated with MPM in asbestos-exposed subjects. Interaction analyses showed that MPM cases and cancer-free controls showed DNAm differences which may be linked to asbestos exposure.

**Abstract:**

Malignant pleural mesothelioma (MPM) is a rare and aggressive neoplasm. Patients are usually diagnosed when current treatments have limited benefits, highlighting the need for noninvasive tests aimed at an MPM risk assessment tool that might improve life expectancy. Three hundred asbestos-exposed subjects (163 MPM cases and 137 cancer-free controls), from the same geographical region in Italy, were recruited. The evaluation of asbestos exposure was conducted considering the frequency, the duration and the intensity of occupational, environmental and domestic exposure. A genome-wide methylation array was performed to identify novel blood DNA methylation (DNAm) markers of MPM. Multiple regression analyses adjusting for potential confounding factors and interaction between asbestos exposure and DNAm on the MPM odds ratio were applied. Epigenome-wide analysis (EWAS) revealed 12 single-CpGs associated with the disease. Two of these showed high statistical power (99%) and effect size (>0.05) after false discovery rate (FDR) multiple comparison corrections: (i) cg03546163 in *FKBP5,* significantly hypomethylated in cases (Mean Difference in beta values (MD) = −0.09, 95% CI = −0.12|−0.06, *p* = 1.2 × 10^−7^), and (ii) cg06633438 in *MLLT1*, statistically hypermethylated in cases (MD = 0.07, 95% CI = 0.04|0.10, *p* = 1.0 × 10^−6^). Based on the interaction analysis, asbestos exposure and epigenetic profile together may improve MPM risk assessment. Above-median asbestos exposure and hypomethylation of cg03546163 in *FKBP5* (OR = 20.84, 95% CI = 8.71|53.96, *p* = 5.5 × 10^−11^) and hypermethylation of cg06633438 in *MLLT1* (OR = 11.71, 95% CI = 4.97|29.64, *p* = 5.9 × 10^−8^) genes compared to below-median asbestos exposure and hyper/hypomethylation of single-CpG DNAm, respectively. Receiver Operation Characteristics (ROC) for Case-Control Discrimination showed a significant increase in MPM discrimination when DNAm information was added in the model (baseline model, BM: asbestos exposure, age, gender and white blood cells); area under the curve, AUC = 0.75; BM + cg03546163 at *FKBP5*. AUC = 0.89, 2.1 × 10^−7^; BM + cg06633438 at *MLLT1*. AUC = 0.89, 6.3 × 10^−8^. Validation and replication procedures, considering independent sample size and a different DNAm analysis technique, confirmed the observed associations. Our results suggest the potential application of DNAm profiles in blood to develop noninvasive tests for MPM risk assessment in asbestos-exposed subjects.

## 1. Introduction

Mesothelioma has a long latency period, usually emerging 20–40 years after asbestos exposure [1]. Malignant pleural mesothelioma (MPM) is rare (prevalence 1–9/100,000), but the corresponding annual death toll worldwide is still estimated at about 40,000 [2,3]. Each year, 125 million people are exposed to asbestos, according to a World Health Organization report [4]. The International Agency for Research on Cancer confirmed that all fibrous forms of asbestos are carcinogenic to humans. The main outcome of exposure is mesothelioma, but cancer at other sites, such as respiratory-tract tumors, are moderately frequent [4]. Previous in vitro studies have demonstrated the cytotoxic effects of asbestos fibers [5,6].

A significant association between MPM and asbestos exposure has been reported, showing a clear, increasing trend in the odds ratio (OR) with increasing cumulative exposure among subjects exposed to over 10 fiber/mL-years [7]. Another study reported that the incidence of malignant mesothelioma (MM) was strongly associated with the proximity of one’s residence to an asbestos exposure source [8].

DNA methylation (DNAm) is an epigenetic mechanism involved in gene expression regulation. In particular, dysregulation of promoter DNAm and histone modification are epigenetic mechanisms involved in human malignancies [9].

According to recent papers, both DNAm and genetic alterations may contribute to MPM tumorigenesis [10,11,12,13,14,15]. Whereas the genome remains consistent throughout one’s lifetime, factors like ageing, lifestyle, environmental exposures and diseases can modify DNAm. The adaptive nature of DNAm means that it can be used to link environmental factors to the development of pathologic phenotypes such as cancers. Fasanelli et al. observed an association between exposure to tobacco and site-specific CpG methylation. They also used peripheral blood DNA to evaluate the importance of these epigenetic alterations in the aetiology of lung cancer [16].

There is less information on the mechanisms and clinical outcomes of epigenetic derangements in MPM [17,18,19]. Several studies have evaluated DNAm alterations in MM samples [20,21,22], but few of them focused on DNAm alteration in blood as a circulating marker. Fischer et al. examined serum DNAm of nine gene-specific promoters from MM cases [23]. A more recent paper identified hypomethylation of a single CpG in *FKBP5* in whole blood cells as a predictor of overall survival in MPM cases [13]. Guarrera et al. evaluated methylation levels in DNA from whole blood leukocytes as potential diagnostic markers for MPM and found a differential methylation between asbestos-exposed MPM cases and controls, mainly in genes related to the immune system [11]. The identification of reliable DNAm biomarkers with high sensitivity and specificity for MPM risk assessment would be a major advancement.

This study was undertaken with the goal to identify new biomarkers for MPM risk assessment and to determine if peripheral blood DNAm profiles have any predictive value. The second goal was to evaluate the interaction effect of asbestos exposure with DNAm on MPM risk. Currently, there are no sensitive testing methods available for the screening of asbestos-exposed individuals who are at high risk of developing MPM. Thus, the identification of reliable MPM diagnostic biomarkers in peripheral blood might provide a tool for detecting the disease at an early stage.

## 2. Results

### 2.1. Epigenome-Wide Association Study (EWAS)

**Figure 1:**
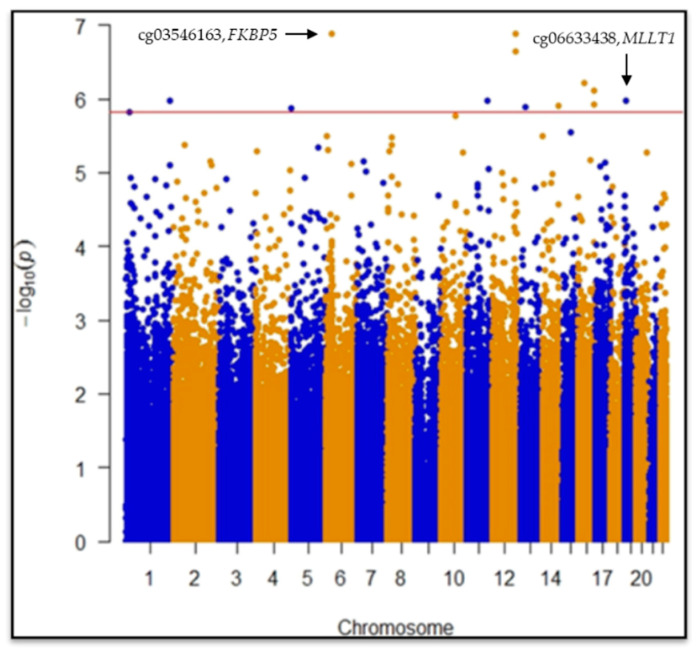
Manhattan plot for EWAS test on 450 k single CpGs. Single-CpG DNAm was used as dependent variable adjusting for age, gender, White blood cells (WBCs: monocytes, granulocytes, natural killer, B cells, CD4+ T and CD8+ T) estimation, population stratification and technical variability. FDR post hoc line highlights statistically significant differences between cases and controls at single CpG level.

CpGs (445,254) passed quality control procedures and were considered for statistical analyses. EWAS revealed two statistically significant differentially methylated single-CpGs between case and control groups: cg03546163 in the *FKBP5* gene (Mean Difference in beta values (MD) = 0.09, 95% CI = −0.12|−0.06, *p* = 1.2 × 10^−7^, *p* = 0.028) and cg06633438 in the *MLLT1* gene (MD = 0.07, 95% CI = 0.04|0.10, *p* = 1.0 × 10^−6^, *p* = 0.049) after False Discovery Rate (FDR) post hoc correction (Figure 1; Table 1).

Another 10 CpGs showed hypo/hypermethylation in MPM considering FDR < 0.05 but not effect size (MD) cut off ≥ |0.05| (Table 1).

Bootstrap was computed to estimate measures of accuracy using random sampling methods. The bias-corrected and accelerated (BCa) bootstrap interval was calculated for cg03546163 in *FKBP5* (95% CIBCa = −0.16|−0.10, z0 = −0.008, a = 0.002) and cg06633438 in *MLLT1* (95% CIBCa = −0.06|−0.1, z0 = −0.011, a = 0.0004) genes, confirming the robustness of the results considering the sample under study.

Statistically significant differences in MD between cases and controls were found in the WBCs estimated (monocytes, *p* = 6.0 × 10^−3^; granulocytes, *p* = 2.2 × 10^−16^; B cells, *p* = 1.1 × 10^−12^; NK cells, *p* = 3.6 × 10^−4^; CD4+ T, *p* = 2.2 × 10^−16^; CD8+ T, *p* = 6.8 × 10^−11^; Naïve CD4T, *p* = 0.012; Naïve CD8T, *p* = 7.0 × 10^−3^).

In order to assess if smoking status, classified as current, former and never-smokers, could modify DNAm profiles, we performed a multivariate regression analysis with the same model used for the discovery phase. No evidence of methylation differences linked to different smoking levels was found for any of the twelve statistically significant CpGs.

### 2.2. Receiver Operation Characteristics (ROC) for Case-Control Discrimination

The baseline model (BM) including age, gender, asbestos exposure and WBCs was compared with BM adding the DNAm levels of cg03546163 or cg06633438. Receiver Operation Characteristics 8ROC9 curves showed a significant increase in MPM discrimination when DNAm information was added in the model (Table 2).

### 2.3. Interaction Analysis

CpG sites and asbestos exposure were considered as predictors of MPM risk in the interaction model. Categorical variables (quantile information) were used considering median values.

We tested the interaction between asbestos exposure and DNAm levels at cg03546163 in *FKBP5* and cg06633438 in *MLLT1.*

Considering cg03546163 in *FKBP5,* DNA hypermethylation and low asbestos exposure levels were used as references, while for cg06633438 in *MLLT1,* DNA hypomethylation and low asbestos exposure levels were set as references (Table 3).

The OR was estimated as the relationship between the combination of single-CpGs DNAm levels and asbestos exposure quantile, and the reference (low median asbestos exposure and hypermethylation status for cg03546163, or hypomethylation status for cg06633438). Age, gender, population stratification, and WBCs were included in the GLM (family = binomial) to adjust the interaction effect.

The relationship between asbestos exposures and single-CpG DNAm levels was evaluated. An increase of one unit of asbestos exposure (rank transformed fibers/mL years) was related to the *FKBP5* gene (β = −0.016, 95% CI = −0.031|−0.001, *p* = 0.044) and *MLLT1* gene (β = −0.014, 95% CI = 0.001|0.026, *p* = 0.035) methylation level variations.

Strong association between asbestos exposure and MPM risk, considering dichotomous distribution of asbestos exposure, was found (OR = 6.11, 95% CI = 3.73|10.20, *p* = 1.8 × 10^−12^). Quartile distribution of asbestos exposure was evaluated to estimate the potential incremental association with MPM risk (1st quartile: used as reference; 2nd quartile: OR = 1.83, 95% CI = 0.93|3.69, *p* = 0.09; 3rd quartile: OR = 6.63, 95% CI = 3.30|13.81, *p* = 2.1 × 10^−7^; 4th quartile: OR = 11.00, 95% CI = 5.26|24.30, *p* = 7.3 × 10^−10^).

### 2.4. Validation and Replication

For the replication and validation approaches, an independent sample of 140 MPM cases and 104 cancer-free asbestos-exposed controls from the same areas were considered, using a targeted DNAm analysis technique.

The direction and magnitude of the association was consistent for cg03546163 and cg06633438 DNAm. Patients showed significantly lower DNAm at cg03546163 (MD = −0.061, 95% CI = −0.087|−0.036, *p* = 4.5 × 10^−6^) and higher DNAm at cg06633438 (MD = 0.024, 95% CI = 0.061|0.013, *p* = 4.0 × 10^−2^) compared with controls. A multivariate analysis confirmed that DNAm at cg03546163 in *FKBP5* and cg06633438 in *MLLT1* were independently associated with MPM detection.

## 3. Discussion

In the present study, we used a whole genome microarray approach to investigate DNAm in WBCs from MPM cases and asbestos-exposed cancer-free controls from a region with a history of asbestos exposure (Piedmont, Italy) [10] in order to identify new noninvasive epigenetic markers related to MPM. The identification of reliable MPM diagnostic biomarkers in peripheral blood might improve risk assessment.

We observed hypomethylation of CpG cg03546163, located in the 5′ UTR region of *FKBP5* gene, in MPM cases compared to controls.

Epigenetic activation of the FKBP Prolyl Isomerase 5 (*FKBP5*) gene has been shown to be associated with increased stress sensitivity and the risk of psychiatric disorders [24]. *FKBP5* is an immunophilin and has an important role in immunoregulation and in protein folding and trafficking. It plays a role in transcriptional complexes and acts as a cotranscription factor, along with other proteins in the *FKBP* family [25]. The suggestion of a possible role of *FKBP5* in the development and progression of different types of cancer has stemmed from several studies. In particular, high protein expression has been linked to either suppression or promotion of tumour growth, depending on tumour type and microenvironment [26,27].

*FKBP5* is involved in the NF-kB and AKT signaling pathways, both of which are implicated in tumorigenesis [28]. Notably, NF-kB appears to be frequently constitutively activated in malignant tumours and involved in the modulation of genes linked to cell motility, neoangiogenesis, proliferation and programmed cell death [29]. An epigenetic upregulation of *FKBP5* could promote NF-kB activation [30]. STAT3-NFkB activity is involved in chemoresistance in MM cells [31], and NFkB was shown to be constitutively active as a result of asbestos-induced chronic inflammation [32].

CpG cg06633438 located in the body region of the *MLLT1* gene was hypermethylated in cases compared to controls.

The *MLLT1* gene encodes the ENL protein, a histone acetylation reader component of the super elongation complex (SEC), which promotes transcription at the elongation stage by suppressing transient pausing by the polymerase at multiple sites along the DNA. In acute myeloid leukemia, *MLLT1* regulates chromatin remodeling and gene expression of many important proto-oncogenes [31]. Yoshikawa and colleagues suggested that mesothelioma may be the consequence of the somatic inactivation of chromatin-remodeling complexes and/or histone modifiers, including *MLLT1* [30].

In mesothelioma patients with short-term recurrence after surgery, frequent 19p13.2 loss was reported. This region encompasses several putative tumor suppressors or oncogenes, including *MLLT1* [32].

**Figure 2:**
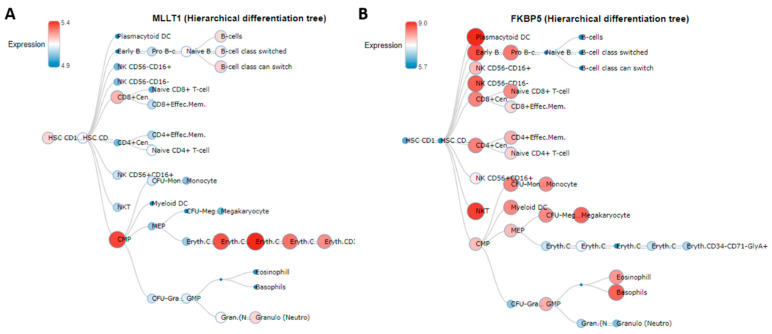
Expression profiles in normal human haematopoiesis. *MLLT1* (**A**) and *FKBP5* (**B**) expression profiles in normal human haematopoiesis as reported in the Blood Spot database (http://servers.binf.ku.dk/bloodspot/, accessed on 26 May 2021).

Interestingly, *MLLT1* and *FKBP5* showed opposite behavior, increasing and decreasing DNAm levels, respectively, in relation to MPM. This finding could reflect the opposite expression profiles of the two genes among all the different subtypes of white blood cells in normal human hematopoiesis, as reported in the Blood Spot database (http://servers.binf.ku.dk/bloodspot/, accessed on 26 May 2021) (Figure 2) [33].

Our interaction analysis showed that considering DNAm levels at *FKBP5* and *MLLT1* genes together with asbestos exposure levels may help to better define MPM risk for asbestos-exposed subjects.

Six single-CpGs showed differential methylation in patients, including those located in *C13orf30, CDC42BPB, ZNF629* and *ALG9* genes; the other six were not annotated to named genes. *ALG9* is a glycogene whose reduced expression has been described during the epithelial-to-mesenchymal transition, an essential process also involved in cancer progression [34]. The *CDC42BPB* gene is ubiquitously expressed in mammals and encodes a serine/threonine protein kinase, a member of the MRCK family [35]. The role of MRCKs in cytoskeletal reorganization during cell migration and invasion has been characterized [36]. The biological function of *C13orf30* and *ZNF629*, a DNA-binding transcription factor, is still to be established.

MPM cases and asbestos-exposed controls showed different proportions of estimated WBCs, which may denote the crucial implication of the immune system. It is known that in cancer, including mesothelioma, the immune system is affected [37], and there is evidence that asbestos directs antigen overstimulation, and that reactive oxygen species production induces functional changes in WBCs [38]. Indeed, in MPM cases, we showed a reduction of estimated CD4+ and CD8+ T lymphocytes, suggesting a weaker adaptive immune system [39]. This may reflect the possible occurrence of functional changes in WBC subtypes in MPM [40,41].

The need for reliable biomarkers is of extreme relevance for a disease such as MPM, which is characterized by the accumulation and persistence of asbestos fibers in the lungs, leading to a long latency period before clear clinical signs of the tumor are detectable. Several biomarkers for early MPM detection (e.g., mesothelin, osteopontin and fibulin-3) have been proposed so far; however, some of them are still under investigation [42]. In this context, DNAm changes in easily-accessible WBCs may provide a useful tool to better assess MPM risk in asbestos-exposed subjects.

Our findings that DNAm levels in single-CpGs in *FKBP5* and *MLLT1* genes are independent markers of MPM in asbestos-exposed subjects suggest the potential use of blood DNAm analysis as a noninvasive test for MPM detection.

Some somatic gene alterations in lung cancer have been linked to tobacco smoke, but few data are available on the role of asbestos fibers: Andujar and colleagues investigate the mechanism of P16/CDKN2A alterations in lung cancer including asbestos-exposed patients. P16/CDKN2A gene inactivation in asbestos-exposed non-small-cell lung carcinoma (NSCLC) cases, a tumor independent of tobacco smoking but associated with asbestos exposure, mainly occurs via promoter hypermethylation, loss of heterozygosity and homozygous deletion, suggesting a possible relationship with an effect of asbestos fibers [43].We observed epigenetic deregulations in the blood of MPM patients compared to that of cancer-free controls, suggesting the potential use of DNAm for risk stratification among asbestos-exposed individuals.

If this observation can be verified in prospectively collected samples, it may be possible to use CpGs methylation to further improve MPM risk estimation for subjects with occupational and/or environmental asbestos exposure.

### Limitation of the Study

Leukocyte DNAm may be a nonspecific marker related to a general, tumour-induced inflammatory status rather than a specific MPM biomarker. Further studies are therefore needed to support our findings.

One main limitation of the functional interpretation of our results is that all our cases had already developed MPM at recruitment: thus, our findings likely reflect disease status rather than being markers of the dynamic processes leading to MPM onset. The lack of MPM tissue from the same subjects also poses major constraints to the functional interpretation of our findings.

Notwithstanding the above limitations, the discrimination between MPM cases and asbestos-exposed cancer-free controls improved when DNAm levels were considered together with asbestos exposure levels.

## 4. Material and Methods

### 4.1. Study Population

Study subjects were part of a wider, ongoing collaborative study, which is actively enrolling MPM cases and cancer-free controls in the municipality of Casale Monferrato (Piedmont Region, Italy). This area was chosen due to its exceptionally high incidence of mesothelioma, caused by widespread occupational and environmental asbestos exposure originating from the Eternit asbestos-cement plant, which was operational until 1986 [44]. Additional MPM cases and cancer-free controls were recruited from other main hospitals of the Piedmont Region (in the municipalities of Turin, Novara and Alessandria). The ongoing collaborative study includes MPM cases diagnosed between incident MPM cases diagnosed between 2000 and 2010 after histological and/or cytological confirmation, and matched controls [45].

The present study included 159 MPM cases and 137 cancer-free controls from a larger case-control study, all of whom had genetic and blood DNAm data [46], good quality DNA at the time of the analyses, and information on asbestos exposure above the background level, as defined in Ferrante et al. [47]. MPM cases and asbestos-exposed cancer-free controls were matched by date of birth (±18 months) and gender. An additional 244 (140 MPM cases and 104 cancer-free controls) independent samples from the same case-control study were included for validation/replication analyses.

Table 4 and Table 5 shows the descriptive characteristics of controls and cases (Min, 1st Q, Median, Mean, 3rd Q and Max) that were considered in the statistical analysis (gender, age, asbestos exposure and WBC estimates: monocytes, granulocytes, natural killer, B cells, CD4+ T and CD8+ T). Asbestos exposure (occupational, environmental and domestic) was normalized considering frequency, duration and intensity. Smoking status (current, former and never smokers) is also explained in Table 6.

Our study complied with the Declaration of Helsinki principles and conformed to ethical requirements. All volunteers signed an informed consent form at enrollment. The study protocol was approved by the Ethics Committee of the Italian Institute for Genomic Medicine (IIGM, Candiolo, Italy).

### 4.2. Exposure Assessment

Information on occupational history and lifestyle habits were collected from all subjects through interviewer-administered questionnaires, which were completed during face-to-face interviews at enrollment. Job titles were coded in two ways according to the International Standard Classification of Occupations [47] and the Statistical Classification of Economic Activities in the European Community.

A cumulative exposure index was computed considering frequency, duration and intensity of asbestos exposure. Occupational, environmental and domestic asbestos exposure were evaluated by an experienced occupational epidemiologist [47], and exposure doses (fibers/mL years) were rank-transformed to remove skewness.

### 4.3. Blood DNAm Analysis and Beta-Value Extraction

DNAm levels were measured in DNA from whole blood collected at enrollment using the Infinium HumanMethylation450 BeadChip (Illumina, San Diego, CA, USA). For blood DNAm analysis (including quality control) please refer to the previous work of the same group [11].

We used the R package ‘methylumi’ to analyze DNAm data. The average methylation value at each locus was computed as the ratio of the intensity of the methylated signal over the total signal (unmethylated + methylated) [48]. Beta-values ranging from 0 (no methylation) to 1 (full methylation) represent the percentage of methylation at each individual CpG locus.

We excluded the following from the analyses: (i) single beta-values with a *p*-value for detection ≥ 0.01; (ii) CpG loci that had missing beta-values in more than 20% of the assayed samples; (iii) CpG loci detected by probes containing single nucleotide polymorphisms (SNPs) with MAF ≥ 0.05 in the CEPH (Utah residents with ancestry from northern and western Europe, CEU) population; and iv) samples with a global call rate ≤ 95%. We also excluded CpGs on chromosomes X and Y.

### 4.4. Batch Effect, Population Stratification and White Blood Cells Estimations

All differential methylation analyses were corrected for “control probes” Principal Components (PCs) to account for variability and batch effects in methylation assays. We used PCs assessed by principal component analysis of the BeadChip’s built-in control probes as a correction factor for statistical analyses of microarray data. This method allows researchers to account for the technical variability in the different steps in DNAm analysis, from bisulfite conversion to BeadChip processing [49].

An individual’s geographic origins may influence DNAm profiles, which could potentially introduce bias. To take this into consideration, we took advantage of the available data from our previous study, which includes a genome-wide genotyping dataset from the same study subjects [50]. When genome-wide genotyping was used to calculate the first PCs, they were shown to correlate with different geographic origins [51].

For each subject, we extracted WBC subtype percentages, estimated based on genome-wide methylation data. This method provides quantification of the composition of leukocytes than can be achieved by simple histological or flow cytometric assessments, with an admissible range of variability [52].

### 4.5. Statistical Analyses

#### 4.5.1. Epigenome-Wide Association Study

An association test was used to analyze the mean differences (MD) in single-CpG methylation between MPM cases and asbestos-exposed cancer-free controls. We performed multiple regression analysis adjusted for age, gender, estimated WBCs (monocytes, granulocytes, natural killer, B cells, CD4+ T and CD8+ T), population stratification (first 2 PCs) and technical variability (first 10 PCs). For multiple comparison tests, a FDR *p* value ≤ 0.05 was considered statistically significant.

Bootstrapping was performed using random sampling methods to estimate the measures of accuracy defined in terms of bias, variance, confidence intervals and prediction error. Bootstrapping can also be applied to control and check the results for stability. The bias-corrected and accelerated (BCa) bootstrap interval was calculated with regard to single CpGs.

ROC for Case-Control Discrimination was implemented, and the AUC metric was applied to estimate the predictive performance of a binary classification (cases/controls). The baseline model (BM) included age, gender, asbestos exposure and WBCs, and was compared with the BM after adding the DNAm levels of statistically significant, single-CpGs at EWAS. AUC differences between BMs before and after the addition of DNAm levels were estimated with DeLong’s test.

#### 4.5.2. Statistical Power

To ensure a study power greater than 99% (two-tailed test at α = 0.05 and β = 0.01), only CpGs with a MD between cases and controls ≥ |0.05| were selected.

Covariates were included step-by-step in a sensitivity analysis to validate the association output considering effect size, standard error, 95% confidence interval and *p* value variations.

Gene set enrichment analyses were carried out on CpGs with a False Discovery Rate *p* value (P_FDR_) ≤ 0.05 to identify pathways that may be affected by MPM-related changes in methylation.

All statistical analyses were conducted using the open source software R (4.0.2).

#### 4.5.3. Interaction Analysis

Logistic regression was used to analyze the relationship between CpGs and asbestos exposure in MPM risk (odds ratio), adjusting for age, gender, SNP PCs and WBCs estimates. Asbestos exposure was classified as above-median or below-median, and CpG methylation was categorized as above-median or below-median.

MPM risk for a given CpG level and asbestos exposure was expressed by ORij, where i indicates the asbestos exposure (below-median or above-median) and j indicates the CpG (above-median or below-median). Considering the direction of the effect, the same approach was used: for hypomethylated CpGs, above-median was used as the reference level, whereas below-median was used for hypermethylated CpGs.

Subjects with below-median asbestos exposure and reference-level CpG DNAm were considered the baseline group, and their MPM risk was coded as OR00 = 1. Interaction was analyzed with respect to both additive and multiplicative models based on the ORs obtained by logistic regression.

Synergistic interaction (positive interaction) implies that the combined action of two factors in an additive model is greater than the sum of their individual effects. Antagonistic interaction, on the other hand, means that when two factors are present in an additive model, the action of one reduces the effect of the other.

Multivariable logistic regression models were used to explore any deviations from a multiplicative model, including asbestos exposure, CpG and the corresponding interaction term (CpG × exposure). All models were adjusted for age, gender, SNP PCs, technical covariates and WBCs estimates. *p*-values < 0.05 were considered statistically significant.

### 4.6. Validation and Replication

DNAm signal validation and replication was done by the EpiTYPER MassARRAY assay (Agena Bioscience). This assay uses a MALDI-TOF mass spectrometry-based method to quantitatively assess the DNA methylation state of the CpG sites of interest [53]. DNA (500 ng) was bisulfite-converted as indicated in Section 4.3.

PCR amplification, treatment with SAP solution and Transcription/RNase A cocktails were performed according to the manufacturer’s instructions, and the mass spectra were analyzed by an EpiTYPER analyzer. The MassARRAY assay cannot discriminate between CpGs located in close proximity in the sequence, so instead, the close neighboring CpGs are analyzed as “Units”, i.e., the measured methylation level is the average of the methylation levels of the CpGs cumulatively analyzed within the Unit. In the case of cg03546163, the measured methylation level is the average between two CpG sites located in very close proximity (Figure S1). For cg06633438, the two adjacent signals were considered, since the results for the model did not differ for effect size, standard error, 95% CI or *p* value (Figure S2).

## Figures and Tables

**Table 1:** Differential DNAm analyses ordered by effect size. Information about single-CpGs, including location-related values and model outputs (effect size, standard error, *p* values).

Probe ID	Chr	Map Position	Gene Symbol	Ucsc Refgene Group	Snp Probe	Effect Size	Standard Error	*p* Value	Fdr	Significance
cg02869235	12	124726864			rs73223527	0.058	0.011	1.3 × 10^−7^	0.028	*§
cg03546163	6	35654363	*FKBP5*	5′UTR		−0.089	0.016	1.3 × 10^−7^	0.028	*§₼
cg02353048	12	124718401				0.033	0.006	2.2 × 10^−7^	0.032	*§
cg06633438	19	6272158	*MLLT1*	Body		0.069	0.014	1.0 × 10^−6^	0.049	*§₼
cg18860329	13	43354421	*C13orf30*	TSS1500		0.050	0.010	1.3 × 10^−6^	0.049	*§
cg19782190	14	103487004	*CDC42BPB*	Body		0.043	0.009	1.2 × 10^−6^	0.049	*§
cg06834916	5	95610				0.037	0.008	1.4 × 10^−6^	0.049	*§
cg09479650	16	85578516			rs4843449	0.037	0.007	1.2 × 10^−6^	0.049	*§
cg26680989	16	85560739			rs80332660	0.036	0.007	7.6 × 10^−7^	0.049	*§
cg25409554	1	234871422				0.034	0.007	1.1 × 10^−6^	0.049	*§
cg01201399	16	30793389	*ZNF629*	Body		0.030	0.006	6.1 × 10^−7^	0.049	*§
cg17283266	11	111717611	*ALG9*	Body		−0.030	0.006	1.1 × 10^−6^	0.049	*§

Control group was set as reference. Adjustment covariates: age, gender, population stratification, WBCs (monocytes, granulocytes, natural killer, B cells, CD4+ T and CD8+ T) estimation and technical variability. *: statistically significant at *p* value < 0.05; §: statistically significant at FDR post hoc adjustments. ₼: statistically significant at beta = 0.01.

**Table 2:** Disease discrimination test considering (AUC) comparison between baseline model and models additionally including single-CpG.

Model	AUC	DeLong’s Test
BM (asbestos exposure, age, gender and WBCs)	0.75	Reference
BM + cg03546163 (*FKBP5*)	0.89	2.1 × 10^−7^
BM + cg06633438 (*MLLT1*)	0.89	6.3 × 10^−8^

Models are shown as baseline model (BM) or BM + Single CpG DNAm. AUC Differences between considered model and BM were estimated with the DeLong’s test.

**Table 3:** Interaction between asbestos exposure and single CpG DNAm on the MPM Odds ratios.

DNAm	Asbestos Exposure	OR	Std. Error	95% CI	*p* Value
cg03546163 (*FKBP5*)					
Hypo	Low	2.79	1.51	1.26|6.33	0.013
Hyper	High	7.21	1.54	3.17|17.27	4.6 × 10^−6^
Hypo	High	20.84	1.59	8.71|53.96	5.5 × 10^−11^
cg06633438 (*MLLT1*)					
Hyper	Low	1.29	1.63	0.70|3.81	0.258
Hypo	High	7.27	1.55	3.17|17.65	5.3 × 10^−6^
Hyper	High	11.71	1.57	4.97|29.64	5.9 × 10^−8^

Reference for cg03546163 in *FKBP5*: hypermethylation and low asbestos exposure levels; Reference for cg06633438 in *MLLT1*: hypomethylation and low asbestos exposure levels.

**Table 4:** Descriptive characteristics of cancer-free control group.

Variable	Controls (Male 100, Female 37)					
	Min	1st Q	Median	Mean	3rd Q	Max
Age	41.60	57.41	65.65	64.59	72.63	90.94
Asbestos exposure	−2.71	−0.97	−0.48	−0.44	0.09	1.73
Monocytes	0.00	0.05	0.06	0.07	0.08	0.26
Granulocytes	0.36	0.54	0.60	0.62	0.68	0.99
Natural Killer	0.00	0.04	0.07	0.08	0.11	0.29
B cells	0.00	0.07	0.09	0.09	0.11	0.19
CD4+ T	0.00	0.10	0.14	0.14	0.19	0.35
CD8+ T	0.00	0.03	0.06	0.07	0.10	0.23

Minimum (Min), First Quartile (1st Q), Median, Mean, Third Quartile (3rt Q) and Maximum (Max) of variables related to cancer-free controls.

**Table 5:** Descriptive characteristics of MPM group.

Variable	Cases (Male 113, Female 50)					
	Min	1st Q	Median	Mean	3rd Q	Max
Age	33.90	61.19	68.68	67.59	75.17	90.80
Asbestos exposure	−2.71	−0.21	0.39	0.37	0.98	2.94
Monocytes	0.00	0.05	0.07	0.08	0.10	0.20
Granulocytes	0.37	0.67	0.74	0.74	0.81	1.03
Natural Killer	0.00	0.02	0.05	0.06	0.08	0.23
B cells	0.00	0.05	0.06	0.06	0.08	0.16
CD4+ T	0.00	0.03	0.07	0.08	0.11	0.22
CD8+ T	0.00	0.00	0.02	0.03	0.04	0.22

Minimum (Min), First Quartile (1st Q), Median, Mean, Third Quartile (3rt Q) and Maximum (Max) of variables related to MPM cases.

**Table 6:** Descriptive characteristics of smoking status stratified by disease.

Smoking Habits	Cases (163)		Controls (137)	
	*n*	%	*n*	%
Current smokers	29	17.79	30	21.90
Former smokers	54	33.13	60	43.80
Never smokers	75	46.01	47	34.31

*n* and % of the three levels of smoking status stratified by disease.

## Data Availability

The methylation of single individuals cannot be published due to informed consent limitations.

## Supplementary Materials

The following are available online at https://www.mdpi.com/article/10.3390/cancers13112636/s1, Figure S1: Location of cg03546163 investigated by EpiTYPER MassARRAY, Figure S2: Location of cg06633438 investigated by EpiTYPER MassARRAY.
