# Supplementary material for: New DNA Methylation Signals for Malignant Pleural Mesothelioma Risk Assessment
Source: Cancers (Basel). 2021 May 27;13(11):2636. doi: 10.3390/cancers13112636 (PMC8199167; doi:10.3390/cancers13112636)
Supplement: Supplementary file 1 [file cancers-13-02636-s001.zip › cancers-1184965-supplementary.pdf]

cancers

MDPI

Article

New DNA Methylation Signals for Malignant Pleural Mesothelioma Risk Assessment

Giovanni Cugliari [1,*], Alessandra Allione [1], Alessia Russo [1], Chiara Catalano [1], Elisabetta Casalone [1], Simonetta Guarrera [2,3], Federica Grosso [4], Daniela Ferrante [5,6], Marika Sculco [7], Marta La Vecchia [7], Chiara Pirazzini [8], Roberta Libener [9], Dario Mirabelli [10,11], Corrado Magnani [5,6,11], Irma Dianzani [7,11] and Giuseppe Matullo [1,11,12,*]

cg03546163

TTTTTGTTTAGGATGAATTAGTTTGGAGAGGAAAGAATATTTTGTTTGTATATTTGT
AGAAGAAGTTATTGTTGTTAAAAGTTAAGTTGGTTTTTTGATGTTTAGTGTGGATAG
GTTGAATAATAATTTATTTTTA**CG**(CpG1)TT**CG**(CpG2)GAGGGTTTATTTTATGTA
AATAGTTGAAAGGAATTGGATAAGATTGTAGTTTTT

Figure S1. Location of cg03546163 investigated by EpiTYPER MassARRAY. Locations of cg03546163 (CpG2, in yellow) and a second CpG site very close (CpG1, in red), investigated by EpiTYPER MassARRAY. Flanking primers are underlined. The CpG sites could not be tested individually due to MassARRAY technology constraints, but had to be tested jointly as a single unit: the methylation level is the cumulative value of all the sites within the CpG unit.

cg06633438

TTTTTTTAGTTTTGGGATTTTGAGATATTAGGATAG**CG**(CpG1)TTATTAATTTAGG
AGTAGTTTTTGTGTTTGTGTGTGTA**CG**(CpG2)TGTG**CG**(CpG3)TTTGTGTGTAGGT
TGGATTTTGATTTTAAAAGGGTTAGGATATGAAAAGAGA**CG**(CpG4)AG**CG**(CpG5)T
TTTAGATTTGAGGAAATATA**CG**(CpG6)**CG**(CpG7)TGGAAGA**CG**(CpG8)TG**CG**(CpG
9)**CG**(CpG10)GGGTT**CG**(CpG11)GGTTTTTTTTTGAGGGAGTGATAAGT**CG**(CpG12
)TGGAAAGTTTTAATATGG**CG**(CpG13)GG**CG**(CpG14)GGGGTGGGGG**CG**(CpG15)A
TAGGATTTGTTTGTTTTAG**CG**(CpG16)GTTTTAT**CG**(CpG17)**CG**(CpG18)TGATAA
TTGAGAGTTTAGAGGGGGA**CG**(CpG19)GAGAGGGAGGGAAGT**CG**(CpG20)GTTAGG
CG(CpG21)T**CG**(CpG22)GGGTATTTAGTTAGGTGG**CG**(CpG23)GGGTTTGAA**CG**(C
pG24)GGGTTT**CG**(CpG25)AAATGTTAAAGAAGAGATAGGTATTGGAGGGATTGTA
GTTATTGGGATATATTTGGTTAGTTGT**CG**(CpG26)AG**CG**(CpG27)ATGG**CG**(CpG2
8)TGGAAAGG**CG**(CpG29)TAGATGGGAGTTGTTTGGTTTGGGGTT**CG**(CpG30)GGA
GGG**CG**(CpG31)GTTTTGTTAGGGGAGGGTGTGGGATTTAGGTTATGGTTTGGTTGG
TTT

Figure S2. Location of cg06633438 investigated by EpiTYPER MassARRAY. Locations of cg06633438 (CpG10, in yellow) and other CpG sites very close (CpGn, in red), investigated by EpiTYPER MassARRAY. Flanking primers are underlined. The CpG sites could not be tested individually due to MassARRAY technology constraints, but had to be tested jointly as a single unit: the methylation level is the cumulative value of all the sites within the CpG unit.
